# The effects of organic nitrates on osteoporosis: a randomized controlled trial [ISRCTN94484747]

**DOI:** 10.1186/1745-6215-7-10

**Published:** 2006-04-26

**Authors:** Sophie A Jamal, Celeste J Hamilton, Dennis Black, Steven R Cummings

**Affiliations:** 1 Department of Medicine, University of Toronto and Division of Endocrinology and Metabolism, St. Michael's Hospital, Toronto, Canada; 2 Department of Epidemiology and Biostatistics, University of California, San Francisco, USA; 3 California Pacific Medical Center Research Institute, San Francisco, USA

## Abstract

**Background:**

Osteoporotic fractures are common and are associated with increased morbidity, mortality and health care costs. The most effective way to moderate increases in health care costs and the sickness and premature death associated with osteoporotic fractures, is to prevent osteoporosis. Several lines of evidence suggest that nitrates, drugs typically prescribed for the treatment of angina, may be effective in preventing postmenopausal osteoporosis.

**Methods:**

We have designed a multicentre randomized controlled trial to determine the effects of nitrates on bone. The trial consists of two studies. The objective of the first study is to determine whether isosorbide mononitrate at 20 mg/day or nitroglycerin ointment at 15 mg/day leads to fewer headaches. The nitrate that is best tolerated will be used in a second study with one main objective: To determine if postmenopausal women with a T-score at the lumbar spine (L1 to L4) between 0 and -2.0 randomized to two years of treatment with intermittent nitrates have a greater increase in spine bone mineral density as compared to women randomized to placebo.

We hypothesize that: 1. Women will report fewer headaches when they are randomized to intermittent nitroglycerin ointment at 15 mg/day compared to intermittent oral isosorbide mononitrate at 20 mg/day, and, 2. After two years, women randomized to intermittent nitrates will have a greater percent increase in lumbar spine bone mineral density compared with women randomized to placebo.

**Discussion:**

We have completed our pilot study and found that transdermal nitroglycerin was associated with fewer headaches than oral isosorbide mononitrate. We are currently recruiting patients for our second main study.

## 1.0 Background

### 1.1 The burden of illness due to osteoporosis

Osteoporosis (OP) is characterized by a reduction in bone mass and disruption of skeletal microarchitecture leading to an increased susceptibility to fracture with minimal trauma. In Canada, one in four women have OP and in 1993 the total expenditure for fractures was in excess of 1.3 billion dollars [1,2]. The average length of stay in an acute care hospital after a hip fracture is three weeks; one of four patients must remain in long-term care institutions for at least one year; and one of three returns home but must depend on other people or devices for mobility. Furthermore, after a hip fracture there is up to a 20% increased risk of mortality [3].

As elderly men and women are the fastest growing group in the world and the incidence of OP fractures increases exponentially with age, the number of men and women with OP fractures is expected to increase dramatically over the next 50 years in Canada and world wide [3]. Assuming current demographic trends continue, the annual incidence of hip fractures worldwide could exceed 21 million in 2050 [4]. The most effective way to moderate increases in health care costs and the sickness and premature death associated with OP fractures, is to prevent OP.

Pharmacologic agents to prevent OP include estrogen replacement therapy (ERT), bisphosphonates (alendronate, risedronate, and etidronate), and selective estrogen receptor modulators (raloxifene). These medications decrease bone resorption. The resultant unopposed bone formation increases bone mineral density (BMD) and decreases fracture rates. Each medication has adverse effects, often resulting in discontinuation. For example, a randomized controlled trial (RCT) of the bisphosphonate alendronate reported that at 2 years, 60% of participants were adherent [5]. Bisphosphonates, if taken improperly, can cause gastrointestinal irritation and esophageal ulceration [6,7]. In addition, bisphosphonates cannot be prescribed for patients with impaired renal clearance, yet these patients are at particularly high risk for fractures [7-10].

Estrogen (with or without a progestin) reduces the risk of all types of fractures by 25–33% but with risks that outweigh its potential benefits for fractures in the vast majority of women [8]. Raloxifene is associated with a 3-fold increase in thromboembolic disease, an increase in hot flushes, leg cramps, leg swelling, and an influenza-like syndrome [9,10].

Pharmacologic treatments are expensive: raloxifene and bisphosphonates cost > $700 Canadian/year, are either unavailable or unaffordable outside of North America and Western Europe, and of uncertain safety when used long-term (> 10 years). The limitations of the current therapies have fuelled interest in alternatives. An optimal agent would be one that decreases bone resorption while also increasing bone formation to have maximal effects on BMD and ultimately fracture, is convenient to take, inexpensive, has minimal adverse effects, is safe for long-term use, and is available world wide. One potential agent is nitric oxide in the form of organic nitrate, the subject of this randomized trial.

This randomized trial consists of two studies. The objective of the first study is to determine whether isosorbide mononitrate (ISMO) at 20 mg/day or nitroglycerin ointment (NTG) at 15 mg/day results in fewer headaches. The nitrate that is best tolerated will be used in a second study with one main objective: To determine if postmenopausal women with a T-score at the lumbar spine (L1 to L4) between 0 and -2.0 randomized to two years of treatment with intermittent nitrates have a greater increase in spine BMD as compared to women randomized to placebo.

### 1.2 Nitric oxide influences osteoclast and osteoblast activity

**Figure 1:**
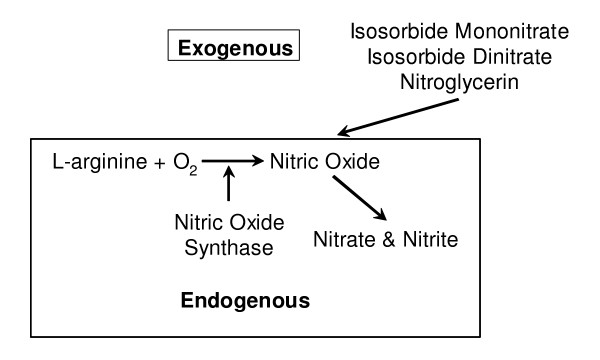
Sources of nitric oxide.

Nitric oxide (NO) is a short-lived free radical involved in the regulation of many physiological processes, including bone remodeling [11,12]. There are three sources of NO (Figure 1). First, NO can be generated by nitric oxide synthase (NOS) from molecular oxygen and the terminal nitrogen of the amino acid L-arginine [12,13]. Second, NO can be generated nonenzymatically from nitrite in the acid environment of the stomach. Third, organic nitrates (e.g. nitroglycerin, isosorbide dinitrate, ISMO) can act as NO donors [14]. In vitro studies consistently demonstrate that NO has a biphasic effect on osteoclast activity and bone resorption [15-19]. Adding a NOS inhibitor to bone cell cultures results in low concentrations of NO and potentiates bone resorption. In contrast, adding NO to bone cell cultures results in high concentrations of NO and decreases osteoclast maturation and bone resorbing activity [20-23]. The effects of NO on osteoblasts and bone formation are less well characterized. Some, but not all, studies have found that low concentrations of NO stimulate osteoblast growth and differentiation and extremely high concentrations inhibit osteoblast growth and differentiation [24].

### 1.3 The effect of nitric oxide on rodent bone

NTG ointment, a NO donor, prevents bone loss in rats [25]. Ovariectomized rats were treated with vehicle, 17-beta estradiol, NTG ointment, or a combination of 17-beta estradiol and NTG. Compared with baseline, treatment with NTG increased BMD in ovariectomized rats (20% ± 3%) to levels similar to those found in sham operated rats (25% ± 2%) and the increase in BMD in ovariectomized rats treated with NTG ointment (20% ± 3%) was greater than the increase in ovariectomized rats treated with vehicle (8% ± 3%) (Table 1). This suggests that nitrates, which act as NO donors, might preserve or even increase bone mass.

**Table 1:** Effect of conjugated estrogen and transdermal nitroglycerin on BMD in ovariectomized rats 25.

Treatment group, n = 5 for all groups	Percent increase in BMD (L2-L4) over 6 weeks
Sham operated	25% ± 2%
Ovariectomized rats	8% ± 3%
Ovariectomized + estrogen	27% ± 5%*
Ovariectomized + transdermal nitroglycerin	20% ± 3%†
Ovariectomized + estrogen + nitroglycerin	22% ± 2%*

*Different than ovariectomized rats at p < 0.005

† Different than ovariectomized rats at p < 0.02

### 1.4 The effect of nitric oxide on bone – human studies

In 1998, we began investigating the relationship between the use of nitrates and BMD in humans using data from the Study of Osteoporotic Fractures (SOF); a multicentre, prospective, observational study of 9704 ambulatory, Caucasian women, aged 65 years and older [26,27]. We hypothesized that women taking nitrates intermittently would have significantly higher bone mass than those who took nitrates continuously. Continuous exposure to organic nitrate causes tachyphylaxis to its vascular effects. Data from the cardiovascular literature report tachyphylaxis to nitrates with increasing frequency of dose [28]. Tachyphylaxis to nitrates may develop in bone; rats given NTG ointment daily for 12 weeks had increases in BMD similar to those with estrogen, yet more frequent administration abolished any beneficial effects, (Table 2) [29].

**Table 2:** Percent change in lumbar spine BMD in ovariectomized rats after 12 weeks of treatment with nitroglycerin (NTG) ointment 29.

Treatment Group (n = 5 for all groups)	Change in Lumbar Spine BMD
Sham operated	6.3% ± 5.3 *
Ovariectomy (OVX)	-2.5% ± 2.0
OVX + estrogen	5.9% ± 3.4*
OVX + 0.2 mg nitroglycerin ointment once a day	6.2% ± 2.8*
OVX + 0.2 mg nitroglycerin ointment twice a day	1.9% ± 2.1
OVX + 0.2 mg nitroglycerin ointment three times a day	-0.2% ± 3.3

We compared hip and heel BMD among nitrate users (391 women) and nonusers (5,827 women) identified by self-report. Women who reported using ISMO, isosorbide dinitrate, or NTG more than once a day, every day, were classified as continuous users (n = 317) and all other women were classified as intermittent users (n = 74). Compared with nonusers, nitrate users were more likely to have risk factors for low BMD [27]. After adjusting for these differences, and for estrogen use, we found that hip BMD was 2.6% higher and heel BMD was 5.3% higher among intermittent nitrate users compared to nonusers and intermittent nitrate users had greater BMD than continuous users at both these sites (Table 3). The results were consistent with our hypothesis that intermittent use of nitrates improves bone mass while continuous nitrate use may lead to tachyphylaxis.

**Table 3:** Differences (mean ± SD) in BMD at the total hip and heel in nitrate users and nonusers. Unadjusted and after adjusting for estrogen use and baseline differences (26).

	Percent difference in BMD (95% CI)	
	
	Daily – nonusers	Intermittent – nonusers
Hip BMD		
Unadjusted	0 (-2.7 to 1.4)	0 (-4.1 to 4.1)
Adjusted*	1.3 (0.14 to 4.1)	2.6 (0.4 to 6.8)
		
Heel BMD		
Unadjusted	-2.6 (-5.3 to 0)	0 (-5.3 to 7.9)
Adjusted*	0 (-2.6 to 2.6)	5.3 (2.6 to 11)

*Adjusted for estrogen use and baseline differences (37), which are current alcohol intake, walks for exercise, physical activity, self-reported health status, falls in the past 12 months, thiazide and nonthiazide diuretic use, inability to rise from a chair independently and frail on physical examination.

There are two potential explanations as to why intermittent nitrate use associated with greater BMD than continuous use. First, women who use nitrates intermittently may have better health and fewer risk factors for low BMD than women who require continuous nitrates. However, adjusting for known for differences in health status did not mitigate the nitrate effect. Second, the findings may be due to chance variation. However the results were robust and statistically significant when we examined BMD at both the hip and the heel.

The average dose of nitrate among women reporting intermittent use was 0.2 mg/day of NTG. This is well below the doses required for angina treatment: a typical single dose is 0.3 mg and daily doses range from 0.3 mg to 0.9 mg. Among the 74 women reporting intermittent use, the type (ISMO, isosorbide dinitrate and NTG), the form (sublingual tablet or spray, oral tablets, sustained release tablets, transdermal patch or ointment), and the dose varied. This suggests an intriguing possibility that nitrates of any type administered intermittently in low doses can increase BMD.

The next step in our program of research was a RCT comparing the effects of placebo and intermittent ISMO on markers of bone turnover in postmenopausal women. We randomly assigned 144 women (≥ 3 years postmenopausal with femoral neck BMD T-scores between 0 and -2.5) to 12 weeks of placebo or intermittent ISMO of 5 mg or 20 mg per day; typically ISMO is prescribed at 20 mg twice a day. We measured changes from baseline in urine N-Telopeptide (NTx), a marker of bone resorption and serum Bone Specific Alkaline Phosphatase (BSAP), a marker of bone formation [30,31].

Our earlier work suggested that the effects of nitrates on bone was a class effect and as such we did not think the type of nitrate we chose would result in substantially different effects on markers of bone turnover [26]. We chose to study ISMO because it is completely and consistently absorbed, does not have a first pass effect, has linear dose-dependent pharmacokinetics, and marked dose-dependent hemodynamic effects [32]. We chose doses of 5 mg and 20 mg; pharmacologic data demonstrate that the threshold of oral activity of ISMO is 5 mg and the maximum response is reached with doses of 20 mg [33]. To prevent tachyphylaxis, we gave ISMO, which is typically administered twice a day, once a day or intermittently [26,29].

We studied markers of bone turnover because substantial changes in markers can occur within 3 months of treatment [34,35]. As such, we were able to assess the potential utility of intermittent ISMO as a preventative agent for OP within a short time. Bone markers, particularly resorption markers, are considered reasonable surrogate end points for fractures [36-38]. A recent study demonstrated that the decrease in NTx observed after three to six months of risedronate therapy was significantly associated with the 75% reduction in vertebral fracture risk at one year and explained about half of the observed reduction in fracture risk [39]. The relationship between formation markers and fractures has not been extensively studied, but some studies suggest an increase in bone formation markers is associated with a decrease in subsequent fractures [40].

**Figure 2:**
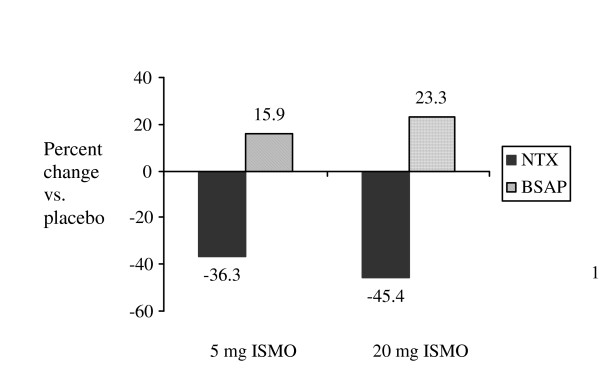
Percent change in urine NTx and serum BSAP in women randomly assigned to 5 or 20 mg of ISMO daily compared with women assigned to placebo.

We found that, compared with placebo, women randomized to intermittent ISMO at 20 mg had a 45.4% decrease in NTx (95% confidence interval [CI]: 25.8 to 64.9) and a 23.3% increase (95% CI: 8.9 to 37.8) in BSAP. Women randomized to intermittent ISMO at 5 mg had a 36.3% decrease in NTx (95% CI: 14.8 to 57.8) and a 15.9% increase in BSAP (95% CI: 1.1 to 30.7) (Figure 2) [30,31]. The decreases in NTx observed with 20 mg of ISMO are similar to those reported with alendronate, risedronate, and estrogen (about 50%) and greater than the 25% decreases reported with raloxifene [40,41]. However, all of the antiresorptive agents concomitantly decrease rates of bone formation. In contrast, we observed that treatment with ISMO resulted in significant increases in BSAP. The decrease in resorption, coupled with the increase in formation, suggests that ISMO may reduce fracture risk to an even greater degree than that seen with the current antiresorptive agents. The only adverse event was headache. Headaches, were more common among women randomized to ISMO (5 and 20 mg groups combined n = 55, 57%) compared with placebo (n = 2, 4%; p = 0.004). Headaches were no more common among women taking 20 mg of ISMO (n = 28) than among women taking 5 mg of ISMO (n = 27; p = 0.7).

Only one other study has examined the effects of nitrates on bone: an open label trial that randomized 16 oophorectomized women, aged 36 to 45, to intermittent NTG ointment (15 mg/day) or oral conjugated estrogen (0.625 mg) [42]. After six months, women taking NTG ointment had a 40% decrease in NTx and 25% increase in BSAP compared with baseline. This study, together with our findings, led U.S. investigators to design a 5 year RCT to study the effects of intermittent NTG ointment compared with placebo on lumbar spine BMD in 200 postmenopausal women. The trial is sponsored by the National Institute of Arthritis and Musculoskeletal and Skin Disease (NIAMS) and is currently recruiting subjects. The RCT we are conducting differs in many important ways from the NIAMS study (see section 2.1 below).

## 2.0 Methods

### 2.1 Study design

This research proposal consists of two placebo controlled trials: a four week pilot study and a 27 month main study. The main study will be preceded by a one week nitrate run-in phase. Both the pilot and main study will recruit healthy postmenopausal women, 50 years of age and older, with lumbar spine BMD T scores between 0 and -2.0. The aim of the pilot study is to determine the best tolerated preparation of nitrate for future studies. We will assess eligibility for the pilot study by questionnaire, focused physical examination, electrocardiogram (ECG) and review of most recent BMD measurements. Subjects (n = 22) will be randomly assigned to intermittent NTG at 15 mg/day and intermittent ISMO at 20 mg/day, each for one week. The order of the treatments will be random, accompanied by a placebo control (identical in sight and smell to the active treatment). In between each treatment there will be a two week washout period. Subjects will record the severity of headaches upon awakening every day for four weeks using a visual analog scale (VAS). We will calculate the mean headache score for each subject over both seven day treatment periods and then the mean headache score considering all subjects for each of the NTG and ISMO treatment periods.

**Figure 3:**
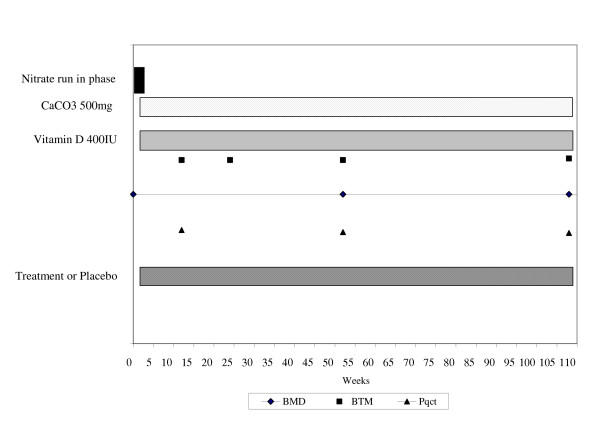
Design of the main trial. *The main trial will be preceded by a one week nitrate run-in phase. Subjects who do not develop headaches during the run-in phase will then enter the main trial. The main trial consists of a 3 month pre-treatment phase with calcium and vitamin D. At 3 months subjects are randomized to treatment of placebo.

The nitrate that is best tolerated will be used in our second, main study, whose primary objective is to assess the effects of intermittent nitrates on spine BMD (L1 to L4). To limit differential drop out due to headaches the main trial will follow from a one week nitrate run-in phase; women who discontinue the nitrate due to headaches will not enter the main trial. We will recruit 280 women and assess eligibility by questionnaire, focused physical examination, ECG, and BMD. We anticipate 17% (48 of 280) of these subjects will be unable to tolerate nitrates due to headaches in the run-in phase, leaving 232 subjects for enrollment into the main study. At the start of the main study all subjects will undergo BMD testing on the same densitometer at the main study centre. We will also determine the total calcium and vitamin D intake, from diet and supplements, using a modified version of the Block food frequency questionnaire. This 34-item questionnaire correlates well (r = 0.76) with the seven day food record and has been validated in postmenopausal women [47-49]. Subjects will be given supplements as required so that all our subjects have an intake of 1500 mg/day of calcium and 800 IU/day of vitamin D. For the first 3 months after study entry, subjects will take only calcium and vitamin D supplements as required (pre-treatment phase). At 3 months, all subjects will have blood and urine samples taken for measurement of bone turnover markers, will undergo peripheral quantitative computed tomography (pQCT) assessments, and will be randomly assigned to placebo or active treatment, in addition to calcium and vitamin D. At 3, 12 and 24 months post randomization, subjects will return to the study centre and provide a fasting blood and second morning urine sample for bone turnover markers; the unused calcium, vitamin D, and study medication will be counted and they will receive a new supply. We will measure BMD and pQCT 12 and 24 months post randomization. The design of the main study is summarized in Figure. 3.

An alternative design would be a single study on the effects of three treatments (placebo, NTG, ISMO) on BMD, bone turnover markers, and pQCT. This design is inferior to our current approach for several reasons. Three treatment arms increases the number of subjects required, and increases recruitment time and study costs. Failure to identify the best tolerated nitrate prior to a larger, longer study may result in a large number of drop outs from adverse events that will reduce study efficiency and increase costs. Furthermore, 3 treatment arms necessitate the manufacturing and administration of both placebo paste and placebo pills, which increases study complexity and costs. Thus, we are well-justified in starting with a small short pilot study and then proceeding with the larger main trial. Three key features of this study that require further elaboration – the patient population, the rationale for the pilot study and nitrate run-in phase, and the outcomes measured – are discussed below.

#### 2.1.1 Patient population: the rationale for studying postmenopausal women

While we recognize that men are also at risk for OP, this RCT will include only postmenopausal women for several reasons. First, studies of nitrates to date have included only postmenopausal women. Second, after menopause in women and age 50 in men, men have higher estradiol levels than women and this difference may influence the effect of nitrates on bone [50]. A third issue is the rate of bone turnover; women, particularly in the first five years after menopause, have higher rates of bone turnover than men with OP [51-53]. These differences in bone turnover rates may lead to heterogeneity with respect to the BMD response to nitrates and may limit the power of our study. In particular, postmenopausal women randomized to placebo may have decreases in BMD while men may not.

#### 2.1.2 The pilot study and nitrate run-in phase

No published studies compare nitrate preparations with respect to the frequency and severity of headaches. In our earlier study, 16 of the 96 (17%) women randomized to ISMO dropped out due to headaches compared with 2 of 48 (4%) women in the placebo [30,31]. All the women in the ISMO group who dropped out due to headaches did so within two days of randomization. The severity of the headaches determined drop out such that a woman with one day of severe headache was five times more likely to drop out than a woman with mild headaches for several days. Headaches were most severe upon awakening and subsided over the day. All headaches resolved within 24 hours of discontinuing the treatment assignment.

In the open label trial discussed earlier women were given 15 mg/day of NTG ointment and no headaches were reported [42]. Although cross-trial comparisons are problematic, this difference suggests that NTG ointment is better tolerated than oral ISMO. One explanation relates to differences in pharmacokinetics; compared with NTG ointment, ISMO results in a much longer exposure to NO [54-57]. Alternative explanations relate to differences in subjects (the open label trial studied young women with surgical menopause who had hot flashes, while we studied healthy older postmenopausal women), or in ascertainment of adverse events (the open label trial relied on self-report whereas we regularly inquired about headaches). Because there is considerable uncertainty about the relationship of nitrate preparation and headaches, our pilot study will focus on which of NTG or ISMO gives the least headaches.

To minimize differential drop out the main trial will be preceded by a one week nitrate run-in phase. All women eligible and willing to enter the trial will be instructed to take the nitrate, identified in the pilot as the best tolerated, for one week (as noted above women who develop headaches do so within 48 hours of taking the drug). Women who do not develop headaches will enter the main trial and, after 3 months of calcium and vitamin D (pre-treatment phase), will be randomized to placebo or treatment.

#### 2.1.3 The intervention: rationale for the nitrate preparation, dose, and frequency of administration

Although several other forms of nitrates (e.g. transdermal patch, sublingual sprays) are available, we chose to study NTG ointment (15 mg/day) and oral ISMO (20 mg/day) because these are the only agents for which data on the safety and tolerability in healthy postmenopausal women exist. Moreover, the only studies that have reported changes in bone turnover markers have used either NTG ointment or ISMO. We will study the 20 mg dose of ISMO as our earlier work found that this dose was associated with the greatest change in bone turnover markers [30,31]. There was no difference in headache incidence among women randomized to 5 mg compared to 20 mg. The 20 mg dose is marketed and readily available; this will be important if we use ISMO in a RCT of nitrates for fracture prevention.

We will study the 15 mg/day dose of NTG ointment. This dose was used in the open label trial of NTG for the prevention of oophorectomy induced bone loss [42]. The 15 mg/day dose was based on dose response studies in rodents: a dose of 0.2 mg/kg/day of NTG was associated with the greatest change in bone turnover markers [25,29,58].

We considered using the NTG patch instead of NTG ointment. The NTG patch has a major disadvantage: it has a long (24 hour) average duration of action and, therefore, may cause tachyphylaxis to the bone effects of nitrate. In contrast, the ointment has a much shorter (6 hour) average duration of action so that once daily administration avoids development of tachyphylaxis. We recognize that ointment is more difficult to apply than a transdermal patch and might stain clothing. However, we have used the ointment in 18 women in our pilot study with no mention by women of messiness or staining of clothes.

We will administer both NTG and ISMO once a day i.e. intermittently. While other dosing frequencies may increase BMD (e.g. continuous dosing, or even once weekly dosing), data from both the cardiovascular literature and our observational study suggest that too frequent dosing (e.g. ISMO 20 mg twice a day or NTG every eight hours) is associated with tachyphylaxis while pharmacologic studies suggest that too infrequent dosing (e.g. less than once a day) results in increased frequency and severity of headaches. Thus, once daily or intermittent administration of nitrates will give the greatest change in BMD and the fewest headaches.

As noted earlier above only one other study (funded by NIAMS) is examining the effects of intermittent nitrates on bone in vivo. Our study differs from the NIAMS study in several important ways: 1. We will determine which of NTG or ISMO is better tolerated in healthy postmenopausal women. This has never been done and is vital, both for the success of our proposal and for the potential clinical use of nitrates in future RCT's and clinical practice. 2. We will determine changes in bone turnover markers in response to nitrates, and 3. We will evaluate changes in bone microarchitecture using pQCT. These latter two outcomes are not being assessed in the NIAMS study, yet turnover markers and microarchitecture contribute substantially to fracture risk [9,62,69].

### 2.2 Trial interventions

#### Pilot study

Subjects will receive NTG ointment; at 15 mg/day (one inch of 2% ointment applied to the upper arm) for one week and ISMO at 20 mg/day for one week. The order of treatments will be randomized. In between each treatment there will be a two week washout. The washout exceeds ten half lives of NTG (one hour) and ISMO (five hours) and will eliminate any carryover effects [54-57]. Subjects who report headaches during the wash out will be excluded from the second treatment phase and considered as drop outs in our analysis. We have considered drop outs in our sample size calculations. Both treatments will be administered with placebo (tablet or paste, as appropriate). Subjects will receive standard instructions on how to take the tablet (at bed time with a glass of water; as was given in our earlier study) and patch (apply it first thing in the morning; as was given in the earlier study).

#### Main study

Subjects who are eligible and willing to participate in the main study will complete a standardized, validated, interviewer administered questionnaire designed to collect general demographics and evaluate factors that have been demonstrated in prospective observational studies to influence levels of bone turnover markers, BMD, and fracture risk [27]. The questionnaire will also assess total daily calcium and vitamin D intake using a modified version of the Block questionnaire. All subjects will be instructed to take the nitrate, identified in the pilot study to be the best tolerated, daily for one week. Subjects who do not develop headaches during the nitrate run-in phase will enter the main trial. The first 3 months of the main trial consist of a calcium and vitamin D pre-treatment phase. We will determine the total daily calcium and vitamin D intake from questionnaire and we will provide calcium carbonate in 500 mg tablets and vitamin D in 400 IU tablets as needed so that the total daily calcium intake for all study subjects will be 1500 mg and the vitamin D intake will be 800 IU; these are the intakes recommended in the recently published guidelines for the prevention and treatment of OP in Canada [70]. Calcium and vitamin D are the mainstay of all treatment regimens for OP and any new agent should be evaluated to assess the additional benefit it would provide. The three month calcium and vitamin D pre-treatment phase will limit any carryover effects of nitrates from the nitrate run-in phase. Further, by providing calcium and vitamin D, we hope to discourage subjects who, after being enrolled in an "OP trial", start taking additional, unreported calcium and vitamin D (co-intervention). Calcium and vitamin D will be prepared and packaged by the study pharmacist, and instructions on how to take the supplements and the PI's office number will be printed on each bottle. Subjects will be given standard verbal instructions by the PI and reminded to take the calcium and vitamin D daily with the morning meal.

All subjects who return to the study centre at three months will undergo pQCT assessments, have blood and urine taken for measurement of bone turnover markers, and be randomly assigned to placebo or active treatment. Subjects will receive a 3 month supply of study medication, calcium, and vitamin D, and will receive standard verbal and written instructions on how to take the medication, calcium, and vitamin D. At 3 months post randomization, subjects will return to the study centre and provide blood and urine samples for bone turnover markers; unused calcium, vitamin D, and study medication will be collected and counted, and subjects will be given a nine month supply of study medication, calcium, and vitamin D. At 12 and 24 months post randomization, subjects will visit the study centre and provide blood and urine samples for bone turnover markers; the unused calcium, vitamin D and study medication will be counted (at the 12 month post randomization visit we will provide a 12 month supply of calcium, vitamin D and study medication) and we will obtain BMD and pQCT assessments (Figure. 3).

### 2.3 Study subjects

The pilot and main study have identical inclusion and exclusion criteria.

#### Inclusion criteria

1. Women aged 50 and older. 2. Lumbar spine BMD (L1 to L4) T score between 0 and -2.0. 3. ≥ 3 years postmenopausal.

#### Exclusion criteria

1. Prior low trauma hip or vertebral fracture; these subjects have OP and require treatment. 2. Total hip or femoral neck T score of < -2.0; these subjects either have OP and require treatment, or are at increased risk of developing OP over the course of the main study. 3. Bone disorders other than osteopenia (e.g. hyperparathyroidism or Paget's disease); these subjects require treatment. 4. Treatment within six months of study entry with androgen, calcitonin, estrogen, progesterone, fluoride in a tablet form, raloxifene, tamoxifen, etidronate, prednisone or an equivalent at 5 mg/d for 12 months or greater, lithium or anticonvulsants. These agents can alter levels of bone turnover markers for up to six months. 5. Alendronate or risedronate use for at least four weeks, within the last three years. These agents may influence bone remodeling for up to three years. 6. Current treatment with nitrates. 7. Systolic blood pressure of ≤ 100 mm Hg or diastolic blood pressure ≥ 110 mm Hg at the baseline screening examination. 8. Abnormal ECG at the baseline screening examination. 9. History of myocardial infarction, angina, valvular or congenital heart disease. 10. Disabling conditions that may interfere with follow-up visits. 11. Inability to give informed consent. 12. Migraine headaches; nitrates can exacerbate migraines. 13. Hypersensitivity to nitrates.

### 2.4 Study outcomes

#### Pilot study

The primary outcome will be mean headache score associated with each of intermittent NTG and ISMO use. Subjects will be given four seven day diaries, with a VAS for each day of both treatment periods and the wash out period. Subjects will rate their headache upon awakening on a daily basis; our previous study demonstrated that subjects randomized to ISMO who developed headaches reported that the headache was most severe upon awakening and gradually subsided over the day [43]. The VAS uses a 100 mm continuous horizontal line with the left pole labeled "no headache" and the right pole labeled "terrible headache"; subjects will be instructed to draw a vertical line intersecting the VAS indicating the severity of their morning headache. We will calculate the mean headache score with intermittent NTG and intermittent ISMO for each subject. Then we will calculate the mean headache score for all subjects when taking ISMO and when taking NTG. The nitrate that gives the lowest mean headache score will be used in our main study. Our earlier work indicates that the nitrate formulation associated with the most severe headaches will result in the greatest number of drop outs [30]. The VAS can detect subtle changes in subjective complaints (i.e. headache), has documented reliability, and has been used in several studies designed to study the effects of NTG ointment on headache in healthy women [56,57].

#### Main study

The primary outcome will be the change from baseline in BMD at the lumbar spine over 24 months among women randomized to treatment compared with women randomized to placebo. BMD will be measured (on the same machine in all subjects) at the lumbar spine (L1 to L4) using a Lunar DPX-L bone densitometer (Lunar Corporation, Madison WI). A single experienced technician, certified by the International Society of Clinical Densitometry (ISCD) and blinded to the treatment assignment, will perform all BMD measurements. The intraclass correlation coefficient for BMD measurements is 0.98 [72]. The PI will be blinded to treatment assignment and will report the BMD. BMD reporting is objective and based on standard ISCD criteria.

We will assess four secondary outcomes in the main study:

##### 1. Change from baseline in total hip BMD

This will provide information about the effects of nitrates on cortical and trabecular bone and ultimately on the ability of nitrates to prevent hip fractures.

##### 2. Change from baseline in bone formation and bone resorption markers

Measuring markers of bone turnover will increase our understanding of the mechanisms by which intermittent nitrates increase BMD (i.e. a decrease in bone resorption and/or an increase in bone formation). We will measure bone turnover markers three months after initiating calcium and vitamin D because we expect a 10% decrease in markers with the use of supplements, and the maximal change in bone markers is typically observed at three months [36]. Thus, we will be assessing any additional effects of intermittent nitrates on markers of bone turnover in women receiving calcium and vitamin D supplements. Urinary NTx will be measured on a second morning urine sample using a monoclonal antibody technique (Osteomark) [73]. The intra-assay variability is 7.6% and the inter-assay variability is 4.0%. Serum BSAP will be measured on a fasting serum sample using a monoclonal antibody technique (Metra Biosystems) [74]. The intra-assay variability is 5.8% and the inter-assay variability is 5.2%. To minimize variability, subjects will be given written and verbal instructions on how to collect the second morning urine sample; for each subject we will collect all the samples (baseline, three, 12 and 24 month post randomization) at the same time, and we will store the blood and urine at -70°C and analyze all the samples together in a single laboratory. We will compare measures of bone turnover markers at 3, 12, and 24 months after randomization using a repeated measure of analysis of variance [75]. By measuring markers at three months we will be able to directly compare these results to the findings in our earlier study. By measuring markers at 12 and 24 months we will be able to evaluate to a limited degree, the question of tachyphylaxis with nitrates. Recall that in our earlier study we found a decrease in markers of bone resorption and an increase in markers of bone formation after 90 days of treatment with intermittent ISMO [30,31]. Measuring markers after one and two years of treatment will allow us to determine if there is still a marked decrease in bone resorption and increase in bone formation; no further changes in markers may indicate that the effects of nitrates on bone is transient.

##### 3. Adverse events

Adverse events will be assessed using a standardized, validated interviewer administered questionnaire on a monthly basis by telephone.

##### 4. Bone microarchitecture

Fracture risk is influenced by both the quantity of bone (assessed by BMD) and quality of bone. Fracture risk is influenced by both the quantity of bone (assessed by BMD) and quality of bone. Indeed, recent reports suggest that up to 40% of fracture risk is explained by decreases in bone quality [5,62]. Our results indicate that nitrates increase bone formation. Exercise and agents, such as PTH, that form bone add bone to the periosteal surface of cortical bone; this may also apply to nitrates. Small increases in the external diameter of bone substantially increase the bending strength of long bones. Unfortunately, bone density by DXA of the complex structures of hip and spine have poor resolution of cortical bone edges and cannot accurately measure the periosteal accretion of cortical bone. In fact, addition of periosteal bone will increase the diameter and, therefore, the projected area of a bone. Since BMD is calculated as total bone mineral content divided by bone area, an increase in bone diameter may paradoxically tend to decrease BMD! Therefore, it is essential to use a measurement that precisely measures cortical dimensions in tubular bone.

Cross-sectional moment of inertia is one of the best biomechanical measurements of the strength of long bones. Trabecular density also contributes to the strength of many bones with high trabecular content. All of these features can be measured with pQCT [66]. PQCT measures are precise, with a minimum detectable change for volumetric bone density of 0.03 g cm^-3 ^[67,68]. PQCT measurements of volumetric bone density and trabecular bone texture have been used by researchers at McMaster University to demonstrate that reductions in fracture risk induced by hormone replacement therapy are due to deposition of bone at the endocortical surface [67]. Statistically significant responses in density, area and texture were detected within six months when 21 women starting therapy were compared with 32 matched controls. Previous work at McMaster was performed with a second generation pQCT machine. The investigators at McMaster have recently obtained a third generation scanner (Stratec XCT2000) that allows measurements of greater reproducibility in both the radius (nonweight bearing) and the tibia (weight bearing). This is particularly relevant with regard to NO as in vitro studies demonstrate that the nitric oxide synthase gene promoter is activated in response to shear stress (equivalent to weight bearing in humans) [68]. Thus, increased levels of NO that results in decreased bone resorption may be one mechanism by which weight bearing prevents bone loss. Our study will test this hypothesis by comparing pQCT measurements in the radius (nonweight bearing) and tibia (weight bearing) among women randomized to nitrates and placebo.

### 2.5 Sample size

#### Pilot study

We wish to detect at least 15 units difference in headache score using the VAS between the intermittent NTG and ISMO groups, which is consistent with a modest effect [57]. We assumed a power of 90% and two sided alpha of 0.05, a standard deviation of 27.78 (based on studies using the VAS to report headaches in healthy women), [56,57] and a small correlation of 0.5 among the repeated measurements [77]. Based on these assumptions, the number of subjects needed for comparing (paired) the mean headache scores for two groups is 20 subjects (Table 4A). We will increase this number by 10%, to account for women who develop headaches that continue into the washout period and cannot go on to the second treatment. Note that this is a conservative approach, in our earlier study all women who developed headaches reported that when they stopped the treatment the headache resolved within 24 hours. Based on these assumptions, we will require 22 subjects for our pilot study.

**Table 4A:** Formula used for sample size calculation for pilot study.

*n *= (*Z*_1-*α*/2 _+ *Z*_1-*β*_)^2 ^{1 + (*n *- 1)*ρ*}/(*n*Δ^2^), where *n *= 7, *ρ *= 0.2, Δ = , *β *= 0.1.

#### Main study

Our primary hypothesis is that, after 27 months, we will observe a clinically important and statistically significant increase in lumbar spine BMD, expressed in percent change from baseline, among women randomized to treatment compared with placebo. Our sample size calculation is based on t-test and will allow us to detect a 2% difference in spine BMD; 2% is the minimal difference that would justify nitrate treatment as this is the minimal magnitude of BMD change observed with other accepted osteoporosis treatments [5,61,62,78,79]. The standard deviation of change with a 2% difference in BMD ranges from 4.5% to 6.8% [5,59,62,80,81]. Assuming a standard deviation of change of 4.5%, a two sided alpha of 0.05 and a power of 0.90 we require 107 subjects per group [81].

The main trial will be preceded by a nitrate run-in phase and only women who complete the run-in phase will be entered into the main trial (see section 2.1aii). Our previous data indicate that 16 of 96 (17%) women randomized to ISMO dropped out due to headaches and 6 of the 144 (4%) women did not return to the study centre after randomization and were lost to follow up [42]. Thus we will "over recruit" by 25% to allow for a 17% drop out in the run-in phase due to headaches and a 8% loss to follow up during the main trial. Thus, we will recruit 280 subjects of whom we anticipate that 232 subjects will complete the run-in phase and enter the main trial (116 subjects per group). This sample size will ensure that we observe a clinically important and statistically significant difference between the active treatment and placebo group. Our sample size gives us adequate power to test our secondary hypotheses (Table 4B).

**Table 4B:** Required sample size to test all secondary hypotheses in main study.

Hypothesis	Clinically Important Difference	Standard Deviation of Change	Subjects, per group
Hip BMD will be higher in treatment group*	2%	4.5% (5, 59, 62, 80, 81)	107
NTx will lower in treatment group†	15% (69, 87–89)	28% (30, 35)	105
BSAP will be higher in treatment group†	15% (69, 87–89)	25% (30)	105
Headaches due to nitrates will be higher in treatment group**	20% (30)	5% (30)	36
Trabecular bone density by pQCT will be higher in treatment group**	10 mg/cm^3 ^(67, 70)	7.5 mg/cm^3 ^(67, 70)	31

* Based on a t-test, assuming a two sided alpha of 0.05 a power of 0.90 and a standard deviation of change in BMD measurements of 4.5%.

† Assuming a repeated measures analysis of variance (75).

** Based on a Student's t test, assuming a two sided alpha of 0.05, a power of 0.90.

The estimated 17% drop out rate from headaches in the run-in phase is conservative as the pilot study will identify the best tolerated nitrate. We anticipate that less than 1% of women will drop out from headaches during our main trial based on the fact that in our earlier trial the majority of the women who dropped out due to headaches did so within the first 48 hours.

### 2.6 Data analysis

Our main analysis will be intention to treat [86]. There will be no subgroup or interim analysis.

#### Pilot study

We will use a paired t test to examine for differences in headache score when subjects are randomized to intermittent ISMO compared to the same subjects randomized to intermittent NTG.

#### Main study

For all of the outcomes in our main study we will compare differences, at 27 months, between the intermittent nitrate group and the placebo group. To determine the percent change in total lumbar spine and hip BMD we will use a Student's t test. We will aim to have follow-up measurements on 100% of subjects randomized and plan to do an intention to treat analysis including measurements. To determine the percent change in BSAP and NTx we will use a repeated measures analysis of variance, after calculating the percent change in BSAP and NTx for each subject (see Table 4C). To determine if the incidence of headaches is higher in the intermittent nitrate group compared to the placebo group we will use a Student's t test. We will also use a Student's t test to determine if trabecular density by pQCT is higher in the intermittent nitrate group compared to the placebo group (Table 4B).

**Table 4C:** Method for calculated percent change in BSAP and NTx

We will calculate the percent change for BSAP* for each participant as:

Then we will average the percent change over all study participants

*We will perform identical calculations for NTx.

### 2.7 Trial management

This is be a multicentre trial that will recruit from five sites: The University Health Network, St. Michael's Hospital, Women's College Ambulatory Care Centre, Sunnybrook Health Sciences Centre, and the Hamilton Health Sciences Centre at McMaster University. As well, we will confer with national and international experts on the conduct of the trial and the interpretation of our data.

All adverse events and significant medical conditions will be recorded and faxed to the committee for adjudication. Subjects will be discontinued from the study if they develop clinical fractures, if their BMD falls below -2.5 at one-year, or if they develop medical conditions that necessitate starting nitrates (e.g. developing angina).

## 3.0 Discussion

We successfully completed our pilot study in August 2005. We found that headaches were significantly lower when women were randomized to nitroglycerin ointment compared with oral isosorbide mononitrate. As a result, the main study is assessing the effects of nitroglycerin ointment on bone mineral density. To date, we have screened 310 subjects, 77 have entered the one week nitrate run in phase, 25 women dropped out after the one week nitrate run-in phase (19 of these due to headaches) have dropped out and 52 women are still participating in the trial.

Nitrates have several advantages over the medications currently used to prevent and treat OP. Unlike ERT, there have been no reports of an increased risk of cardiovascular disease or breast cancer with long-term use of nitrates [43-46]. Compared with bisphosphonates and raloxifene, nitrates are generally more available world wide, more convenient to take, and less expensive. Clearly, further studies of nitrates are essential.

The value of nitrates for prevention of fracture will require a randomized blinded trial with a fracture outcome. We plan to conduct an international trial comparing intermittent nitrates with standard pharmacologic therapy to prevent fractures in women with OP. Prior to the RCT, we must determine the form of nitrate that is associated with the least severe headaches and also determine if intermittent nitrates can increase spine BMD. These are the main objectives of this trial.

## Competing interests

The author(s) declare that they have no competing interests.

## Authors' contributions

All 4 authors have made substantial contributions to the study conception and design, acquisition of data and analysis and interpretation of data. Each author has been intimately involved in drafting and editing the manuscript and approves the final version for publishing.
